# High-Throughput Screening of Myometrial Calcium-Mobilization to Identify Modulators of Uterine Contractility

**DOI:** 10.1371/journal.pone.0143243

**Published:** 2015-11-24

**Authors:** Jennifer L. Herington, Daniel R. Swale, Naoko Brown, Elaine L. Shelton, Hyehun Choi, Charles H. Williams, Charles C. Hong, Bibhash C. Paria, Jerod S. Denton, Jeff Reese

**Affiliations:** 1 Department of Pediatrics, Division of Neonatology, Vanderbilt University School of Medicine, Nashville, Tennessee, United States of America; 2 Department of Entomology, Louisiana State University Agricultural Center, Baton Rouge, Louisiana, United States of America; 3 Department of Pediatrics, Division of Critical Care Medicine, Vanderbilt University School of Medicine, Nashville, Tennessee, United States of America; 4 Department of Cell and Developmental Biology, Vanderbilt University School of Medicine, Nashville, Tennessee, United States of America; 5 Department of Medicine, Division of Cardiovascular Medicine, Vanderbilt University School of Medicine, Nashville, Tennessee, United States of America; 6 Department of Pharmacology, Vanderbilt University School of Medicine, Nashville, Tennessee, United States of America; 7 Department of Anesthesiology, Vanderbilt University Medical Center, Nashville, Tennessee, United States of America; Medical College of Wisconsin, UNITED STATES

## Abstract

The uterine myometrium (UT-myo) is a therapeutic target for preterm labor, labor induction, and postpartum hemorrhage. Stimulation of intracellular Ca^2+^-release in UT-myo cells by oxytocin is a final pathway controlling myometrial contractions. The goal of this study was to develop a dual-addition assay for high-throughput screening of small molecular compounds, which could regulate Ca^2+^-mobilization in UT-myo cells, and hence, myometrial contractions. Primary murine UT-myo cells in 384-well plates were loaded with a Ca^2+^-sensitive fluorescent probe, and then screened for inducers of Ca^2+^-mobilization and inhibitors of oxytocin-induced Ca^2+^-mobilization. The assay exhibited robust screening statistics (Z´ = 0.73), DMSO-tolerance, and was validated for high-throughput screening against 2,727 small molecules from the Spectrum, NIH Clinical I and II collections of well-annotated compounds. The screen revealed a hit-rate of 1.80% for agonist and 1.39% for antagonist compounds. Concentration-dependent responses of hit-compounds demonstrated an EC_50_ less than 10μM for 21 hit-antagonist compounds, compared to only 7 hit-agonist compounds. Subsequent studies focused on hit-antagonist compounds. Based on the percent inhibition and functional annotation analyses, we selected 4 confirmed hit-antagonist compounds (benzbromarone, dipyridamole, fenoterol hydrobromide and nisoldipine) for further analysis. Using an *ex vivo* isometric contractility assay, each compound significantly inhibited uterine contractility, at different potencies (IC_50_). Overall, these results demonstrate for the first time that high-throughput small-molecules screening of myometrial Ca^2+^-mobilization is an ideal primary approach for discovering modulators of uterine contractility.

## Introduction

The uterine myometrium is a therapeutic target for the inhibition of uterine contractility to delay the early onset of labor, or the stimulation of uterine contractility to induce labor or control postpartum hemorrhage. Current therapeutics used to inhibit premature contractions (termed tocolytics) are associated with detrimental off-target side effects for both infant and mother when used to maintain pregnancy beyond 24–72hrs [1–3]. Conversely, women who develop postpartum hemorrhage as a result of uterine atony and unresponsiveness to contractile agonists (termed uterotonics), frequently require emergency surgical intervention (*i*.*e*. hysterectomy). These issues have led to the growing recognition that novel tocolytic and uterotonic agents are urgently needed.

An increase in intracellular Ca^2+^ in uterine myometrial (UT-myo) cells is the final common pathway controlling myometrial contractions [1,4]. Myometrial contractions during labor are dependent on binding of OT and prostaglandins (PGs) to their receptors on UT-myo cells, stimulating the release of Ca^2+^ from intracellular stores, promoting Ca^2+^-entry into cells, and triggering coordinated, synchronous myometrial contractility [1,5–10]. Current tocolytic therapeutics used to inhibit preterm labor includes: atosiban (an OT-receptor antagonist), indomethacin (an inhibitor of PG-synthesis), ritrodine (a beta-adrenergic agonist), nifedipine (a Ca^2+^-channel blocker), and magnesium sulfate (intracellular-Ca^2+^ effector) [11–14]. Conversely, oxytocin remains the preferred uterotonic agent for labor induction and prevention of postpartum hemorrhage [15].

Current research tools used for small-scale discovery or testing of tocolytics and uterotonics have utilized either: 1) computational modeling [16,17], 2) fluorescence-based Ca^2+^-assay [18–20], 3) recording Ca^2+^-channel currents using whole-cell patch clamp technology [19,21], 4) collagen gel contraction assay [22,23], 5) oxytocin receptor (OTR) assays [24], 6) *ex vivo* measurements of myometrial tension/contractility [25–31] [formerly referred to as oxytocic bioassay [24]], or 7) *in vivo* measurements of intrauterine pressure [32–34]. However, to our knowledge there are no reports of large-scale screening for the discovery of new tocolytic or uterotonic compounds.

High-throughput screening (HTS) of small-molecule libraries is the standard approach used in the pharmaceutical industry to discover new lead compounds for drug development. Although a majority of drug discovery efforts are centered around HTS for modulators of molecularly defined, single drug targets, these often ignore the complexity of cell signaling pathways that underlie important physiological processes. HTS of calcium mobilization utilizing fluorescent Ca^2+^-sensitive probes circumvents this limitation and allows testing of large collections of compounds to identify both agonists and antagonists in a single screen [35]. The benefit of using primary cells in HTS lies in their retention of many *in vivo* functions and endogenous expression of mechanisms/targets of interests [36]. However, primary cells must be proven reproducible for reliable use in HTS. Here we report the development and validation of a fluorescence-based Ca^2+^-assay using primary mouse UT-myo cells for identification of uterotonics and tocolytics. Functional annotation analysis of identified hit-compounds provided insight into the pharmacological classes and protein targets that affect both native and OT-induced myometrial Ca^2+^-mobilization. In a secondary screen using an *ex vivo* isometric contractility assay, we show the ability and potency of four hit-antagonists to dampen uterine myometrial contractions. Overall, these findings demonstrate that a robust OT-induced Ca^2+^-mobilization assay can be utilized for screening large compound collections to identify modulators of uterine contractility.

## Materials and Methods

### Isolation of Murine Uterine Myometrial (UT-Myo) Cells

All animal experiments were approved by the Vanderbilt University Institutional Animal Care and Use Committee and conformed to the guidelines established by the National Research Council Guide for the Care and Use of Laboratory Animals. Adult (8–12wk) CD1 wild-type (Charles River Laboratories) mice were housed in 12h light: 12h dark cycle, with free access to food and water. Timed-pregnancies were performed, and the presence of a vaginal plug was considered day 1 of pregnancy, with the time of expected delivery on d19.5. Mice were euthanized by cervical dislocation under a lethal dose of isoflurane. Upon removal from d19 pregnant mice, uteri were placed into ice-cold Hank’s Buffered Saline Solution (1X HBSS, without Ca^2+^ or Mg^2+^), and cut longitudinally along the mesometrial border. After removal of fetuses, placentas, amniotic and endometrial membranes, the myometrium was cut into ~1mm^3^ pieces and digested in 0.2% Type-II Collagenase (Worthington Biomedicals) in HBSS for 45–60 min at 37°C in 5% CO_2_ atmosphere. Following tissue digestion, cells were suspended in complete media (phenol-free DMEM supplemented with 10% fetal bovine serum (FBS), 25 mM HEPES, 100 U/ml penicillin-streptomycin) then filtered through 100-micron nylon cell strainers. Isolated cells were centrifuged at 1000rpm for 10 min, then resuspended in complete media and subjected to a differential attachment technique [37] to selectively enrich for uterine myocytes. Specifically, UT-myo cells were plated in 150mm cell culture dishes for 2hr at 37°C in 5% CO_2_ atmosphere, during which non-myocytes (mostly fibroblasts) attached to the bottom of the cell culture dish. The supernatant, containing the slowly adhering uterine myocytes, was collected and transferred to 150mm cell culture dishes. After 24hrs the media was changed. The cells became near-confluent after 48hrs, at which time the cells were dissociated using 0.25% Trypsin-EDTA. Once resuspended in complete media, cells were plated in either 4-well chamber slides (Tissue-tek) for characterization of the cell population or clear-bottom, black-walled and poly-D-lysine-coated 384-well plates (Grenier Bio-One) for high-throughput screening Ca^2+^-mobilization assays.

### Primary mouse UT-Myo cell characterization

Immunofluorescent labeling of cells for smooth muscle α-actin and calponin antibodies was performed to access the purity and homogeneity of our UT-myo cell cultures, similar to Tribe *et al*. [38]. After 24 hours, UT-myo cells on chamber slides were washed three times using 1X Phosphate Buffered Saline (PBS) prior to fixation using 4% paraformaldehyde (PFA) for 30 minutes. Additionally, frozen sections (8-micron thickness) of whole mount uterus was collected from day 19 of mouse pregnancy to compare to the isolated UT-myo cell population. Whole-mount uterine sections were fixed with 4% PFA for 2 hours at room temperature, followed by sucrose infiltration and embedding. After washing three times with 1XPBS, blocking solution (10% goat serum, 0.25% Triton X-100 in PBS) was applied for 1 hour at room temperature, followed by an overnight incubation at 4°C in the primary antibody, either: smooth muscle actin (1:250 dilution; Sigma, A 2547) and/or calponin. (1:100 dilution; Abcam ab46794). After washing with 1X-PBS, samples were incubated with fluorescent-labeled secondary antibodies (1:2000; Invitrogen A-11004 and/or A-11008) and DAPI for 3 hours at room temperature. Slides were coverslippped using Aqua Polymount (Polysciences, Inc), and visualized under a fluorescent microscope.

### Ca^2+^ Mobilization Assay Development and Pilot HTS

Assays were performed in the Vanderbilt Institute of Chemical Biology High-Throughput Screening Facility. After 24hr attachment, cells were washed twice with wash buffer [1X HBSS Ca^2+^ and Mg^2+^ containing 20mM HEPES and 2.5mM probenecid (Sigma-Aldrich)] using an ELx405™ (BioTek) automated microplate washer. A final aspiration step resulted in 20uL residual volume. The cells were loaded with 20uL of 2X loading buffer [wash buffer containing Fluo-4AM (Invitrogen/Molecular Probes) and 0.023% (w/v) pluronic acid (Invitrogen)] per well using a Multidrop™ Combi (Thermo Fisher Scientific) reagent dispenser. Probenecid was used to block the active transport of the Fluo-4AM out of the cells, while pluronic acid was used to stop Fluo-4AM breakdown by external esterases. After 1hr incubation at 37°C (5% CO_2_ atmosphere), cells were washed twice with wash buffer (ELx405™) again resulting in 20uL residual volume. The cells were transferred to a Functional Drug Screening System (FDSS 6000; Hamamatsu) to measure baseline fluorescence for 20sec (1Hz; Ex480:Em540) followed by the “compound addition” of 20uL of 0.2% DMSO (0.1% final concentration; Sigma-Aldrich) using the FDSS’ integrated pipettor and continuous measurement of fluorescence for an additional 40 seconds. The plate remained at room temperature, protected from light, for 30min to allow de-esterification/hydrolysis of the Fluo-4AM. Afterwards, the fluorescence was measured by the FDSS for 20 sec (1Hz; Ex480:Em540) prior to recording transient intracellular calcium mobilization for 140 sec following exposure to the “OT addition”: OT (O6379; Sigma-Aldrich,) or vehicle in 10μL wash buffer, for a final concentration of 100 pM to 100 μM. Non-linear regression analyses were performed to generate concentration response curves (CRCs) using a four-parameter logistical equation in Prism 6 (GraphPad). Signal (OT) was normalized to background Max-Min relative fluorescent units (RFU) to determine optimal cell plating density and Fluo-4AM concentration. Comparisons of fit were performed to determine if the non-linear fit lines between the cell densities and Fluo-4AM concentrations were significantly different (p<0.05). Two-way analysis of variance was used to determine significant differences between each cell density and Fluo-4 concentration examined.

#### Oxytocin CRC to determine EC_80_

After establishing the optimal cell plating density (8,000 cells/well) and Fluo-4AM concentration (4μM final concentration), additional Ca^2+^-mobilization assays were performed to obtain the EC_80_ for OT. During the compound addition, rows A, H and P received only DMSO (0.1% final concentration); while 13-point three-fold dilutions of OT (starting at 100uM final concentration) were performed in the remaining rows. For each well, the mean baseline value (MBV) for Ca^2+^-fluorescence was calculated during the 0–19 sec timeframe. The max RFU was calculated from the 20–140 sec timeframe, and then subtracted from the baseline. An average Max-MBV RFU was calculated for each OT concentration. Data were analyzed using Prism 6. Non-linear regression analyses were performed to generate concentration response curves and to determine the OT EC_80_ value for each experimental day (range = 0.82μM to 8.68 μM) of: 1) checkerboard assays for Z´ determination; 2) pilot screen; and 3) confirmation of “hit”-compounds.

#### Checkerboard Assays for Z´-factor determination

‘‘Checkerboard” assays were performed on 3 separate days to determine the well-to-well uniformity within a plate and day-to-day reproducibility. Calcium-mobilization assays were performed as described above. During the compound addition, every alternate well received 20μL of either 0.2% (0.1% final concentration) DMSO or 20uM (10μM final concentration) atosiban (Sigma-Aldrich A3480). During the OT addition, the entire assay plate received the OT EC_80_ dose determined on that experimental day using a partial plate (at least 4 columns) of cells. A Z´-factor [39] was calculated for each batch of myometrial cells using:

$\text{Z}\prime \text{factor}\;=1-\frac{3({\text{SD}}_{\text{c}+}+{\text{SD}}_{\text{c}-})}{\left|{\text{M}}_{\text{c}+}-{\text{M}}_{\text{c}-}\right|}$; SD_c+_ and SD_c−_represent standard deviation (SD) for the positive control (DMSO + OT) and negative control (atosiban + OT), respectively. M_c+_ and M_c−_represent the mean for the positive control and negative control, respectively.

#### Assessment of DMSO Tolerance

Small-molecule library compounds are dissolved in the solvent DMSO. Since DMSO could have direct effects on the Ca^2+^-mobilization assay and lead to false-positive hit identification, we examined the assay’s tolerance to DMSO by performing a titration of DMSO ranging from 0.0025% to 1% v/v final concentration. A total of 6 columns were used to examine DMSO tolerance. The DMSO was added during the “compound” addition, followed by OT-EC_80_ addition 30 minutes later. An average Max-MBV RFU was calculated for each DMSO concentration. Data were analyzed using Prism 6. Non-linear regression analyses were performed to generate concentration response curves

#### Pilot Screen for Assay Validation

Four columns in each plate used for HTS were filled in a checkerboard pattern with either DMSO control or atosiban during the compound addition, followed by OT addition to all wells, as described above. This was performed in order to calculate a Z´-factor for each pilot screen compound plate. During the pilot screen, 320 test compounds filled columns 3–22 of the 384-well plate. Test compounds in 40 μL of wash buffer were prepared by transferring 80 nL into a 384-well polypropylene plate using the ECHO 555 acoustic liquid handler (Labcyte, Sunnyvale, CA) from a 10mM stock. Test compounds (10μM final concentration) were added during the compound addition, with OT (EC_80_) added during the OT addition. In this manner, compounds with an inhibitory effect on OT-induced Ca^2+^-mobilization in UT-myo cells might be identified. The MicroSource Discovery Systems Spectrum Collection (2000 compounds), National Institute of Health Clinical Collection I (446) and II (281) compound libraries were screened using a total of 10 microtiter plates.

A “mean high” value was calculated by averaging the Max-MBV RFU from all of the wells that received Vehicle + OT EC_80_. A “mean low” value was calculated by averaging the Max-MBV RFU from all of the wells that received atosiban + OT EC_80_. Percent response was calculated from data collected during the compound addition, using: $\%\;response=\frac{Max-MBV}{MBV}*100$.

### Selection and confirmation of “hit” compounds

A robust Z´-score calculation was used to identify hit-compounds based on whether a test compound’s value was greater than 3 times the mean absolute standard deviations (MADs) away from the median of all the vehicle (VEH) wells.

$$\%response\geq median\;(\%\;{response}_{VEH})\;+\;3\;*\;mad\;(\%\;{response}_{VEH})$$

$$\text{or}\;\%response\leq median\;(\%\;{response}_{VEH})\;-\;3\;*\;mad\;(\%\;{response}_{VEH})$$

Finally, all compounds identified as hits were cherry-picked from library plates for confirmation testing and concentration-response titrations using the Ca^2+^-mobilization assay. Confirmation testing was performed in duplicate at 10 μM concentration to confirm activity all hit-compounds.

### Concentration-response titrations of hit-compounds

We examined the potency of the confirmed hit-agonist and antagonist compounds by assaying compounds at three-fold 10-point titrations starting at 30μM. Compounds were transferred to daughter plates using an ECHO acoustic plate reformatter, and tested as described above for the primary screen. Two compounds were purchased from commercial vendors to compare compound potency to those cherry-picked from the compound library plates: benzbromarone (Sigma B5774) and spiperone (MP Biomedicals 0215207283).

### Functional annotation in PubChem

Each confirmed hit-compound was searched for in the National Institutes of Health (NIH) PubChem database to obtain its compound identification (CID) number and Medical Subject Heading (MeSH) pharmacological classification(s). Each confirmed hit’s CID was uploaded into BioActivity SAR in PubChem. CIDs were clustered by Compound Activity and Active BioAssay Defined Protein Targets.

### Functional *Ex Vivo* Uterine Isometric Contractility Assay

Uterine myometrial samples were obtained from CD1 wild-type mice on day 19 of pregnancy, on at least three different experimental days. Longitudinal strips of uterine myometrium were prepared by cutting the uterus along the mesometrial (vascularized) border, followed by removal of fetal, placental and connective tissue/membranes. Uterine strips of 4mm X 12mm were attached via silk thread to stainless steel hooks connected to a Radnoti^LLC^ force transducer at one end, while the other end of the tissue was anchored to a glass rod at the base of the tissue bath. Preparations were then submerged in a heated and oxygenated (37°C, 95% O2-5% CO2) Radnoti^LLC^ tissue bath containing Kreb’s Bicarbonate Solution (136.7mM NaCl, 4.7mM KCL, 2.5mM CaCl_2_2H2O, 1.5mM MgCl_2_6H_2_O, 1.8mM NaH_2_PO_4_H_2_O, 15mM C_6_H_12_O_6_ and 2.52mM NaHCO_3_). Each strip was placed under 1g tension and allowed to equilibrate in the organ chamber for 60min prior to recording baseline spontaneous contractile activity. Tissue strips that failed to establish a regular amplitude and frequency were excluded from further study. Following the establishment of spontaneous contractions, cumulative doses of either atosiban or confirmed hit-compounds were added to individual organ baths every 10 min. The following hit-compounds tested in the organ bath were purchased from Sigma-Aldrich: atosiban (A3480), benzbromarone (B5774), dipyridamole (D9766) and nisoldipine (N0165); while fenoterol hydrobromide (HBr) was purchased from MP Biomedicals (0215803983). Isometric contractions were recorded using PowerLab/8 SP (ADInstruments) hardware and analyzed with LabChart 7 Pro software (ADInstruments). Contractile activity was assessed by amplitude (cyclic height), frequency (number of contractions/10min) or AUC/sec (area under the curve, which is the sum of the integrals for each contraction divided by the duration of the period assessed). All treatment data were then expressed as a percentage of the baseline spontaneous contractile activity. Data were analyzed using Prism software. Data are expressed as mean±SEM. Non-linear regression analyses were performed to generate CRCs for calculation of IC_50_ and E_max_. Comparisons of fit were performed to determine if the non-linear fit lines between the compounds were significantly different (p<0.05) from DMSO. Two-way analysis of variance was used to determine significant differences between the % response for each concentration of a given compound versus DMSO.

## Results

### Characterization of Primary Mouse Uterine Myometrial Cells

**Fig 1:**
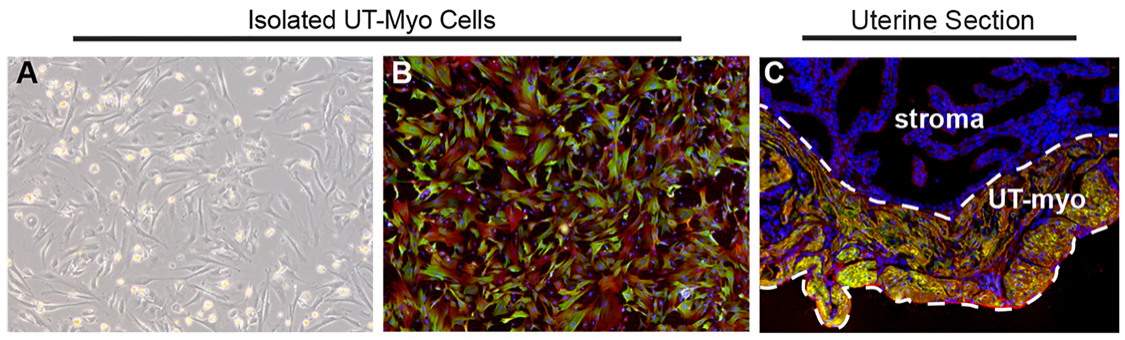
Assessment of primary murine UT-myo cell homogeneity. A. Representative photomicrograph of UT-myo cells prior to dissociation and use in Ca^2+^-mobilization or immunofluorescent staining. Representative photomicrograph of UT-myo cells (B) and uterine myometrium (C) stained with smooth muscle cell markers, alpha-SMA (red) and calponin (green), and DAPI (blue). UT-myo cells and whole-mount uterine tissue were collected from day 19 of mouse pregnancy. The placenta and embryo were removed from whole-mount tissue sections.

The goal of this investigation was to prepare a UT-myo cell culture that was: 1) homogenous and reproducible for HTS; 2) contained properties comparable to myometrium *in vivo*. As shown in Fig 1, the majority of isolated UT-myo cells stained positive for two markers of smooth muscle cells, alpha-SMA and calponin (panel B), similar to that of whole uterine tissue collected from day 19 of mouse pregnancy (panel C).

### Development of a Uterine Myometrial Cell Ca^2+^ Mobilization Assay for HTS

**Fig 2:**
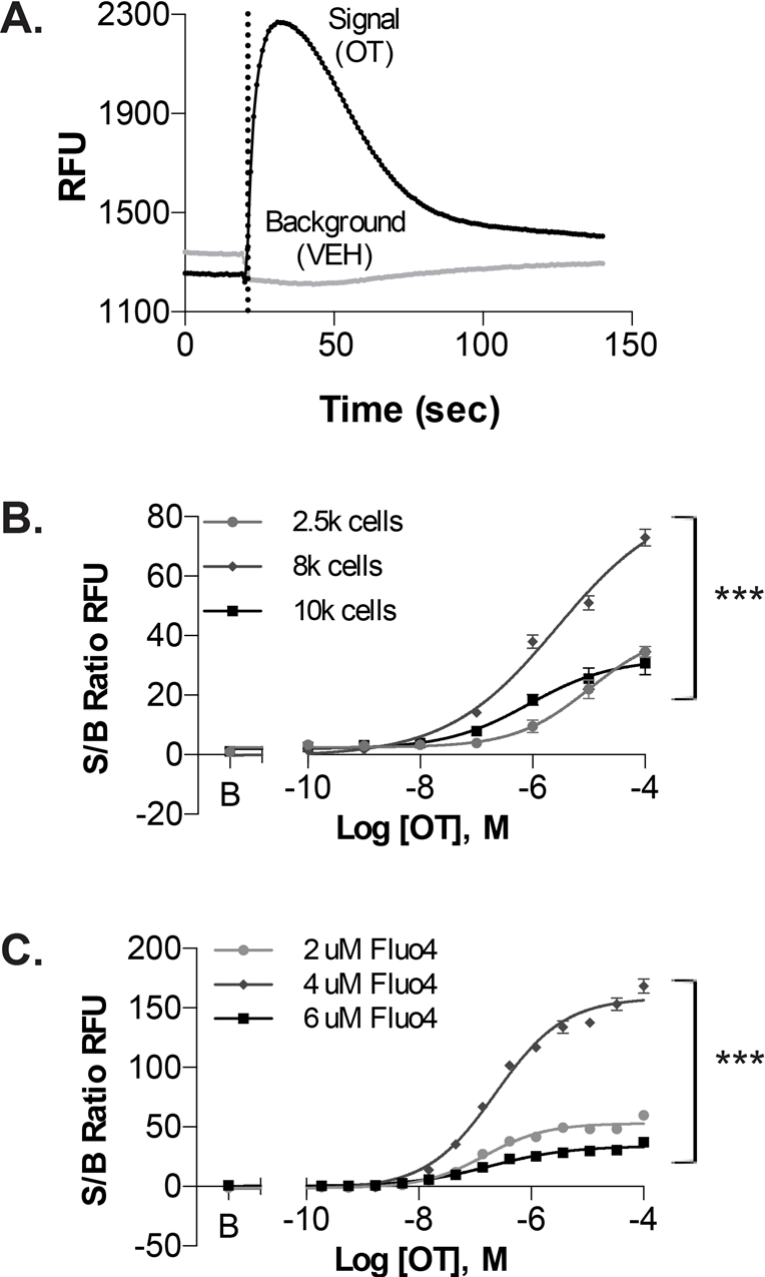
Ca^2+^-mobilization assay using uterine myometrial cells. A. Representative recording of OT-induced Ca^2+^-mobilization from UT-myo cells loaded with Fluo-4AM. Ca^2+^-mobilization was monitored as an increase in Relative Fluorescent Units (RFUs). Dashed line indicates time of OT or vehicle (VEH) addition. Optimal assay conditions were determined by performing cell density gradient (B) and Fluo-4AM concentration response curves (C), from signal-to-background (S/B) ratios of Max-Min RFU obtained from OT and VEH wells, respectively. Non-linear regression was used to fit the data (Mean±SEM; n = 8 well replicates); significant (***p<0.0001) difference between each fit line.

Oxytocin, the most potent endogenous contractile-agonist for induction of labor [40], was selected as the stimulus for intracellular Ca^2+^-release from UT-myo cells. Treatment of UT-myo cells with OT (100pM to 100μM; indicated by dashed line in Fig 2A) resulted in a robust (~15sec to peak) concentration-dependent increase in intracellular Ca^2+^-release (indicated by relative fluorescent units; RFUs). By contrast, treatment with vehicle (0.1% DMSO final concentration) had no effect on intracellular Ca^2+^-release. In order to optimize the Ca^2+^-mobilization assay for mouse primary UT-myo cells, we performed cell density and Fluo-4AM dye concentration gradients (Fig 2B and 2C, respectively). OT CRCs were used to determine optimal assay conditions based on signal (OT) to background (vehicle) ratios. Based on these results, we concluded that 8,000 cells/well loaded with 4μM Fluo-4AM dye in 384-microtiter plates resulted in optimal assay conditions.

**Fig 3:**
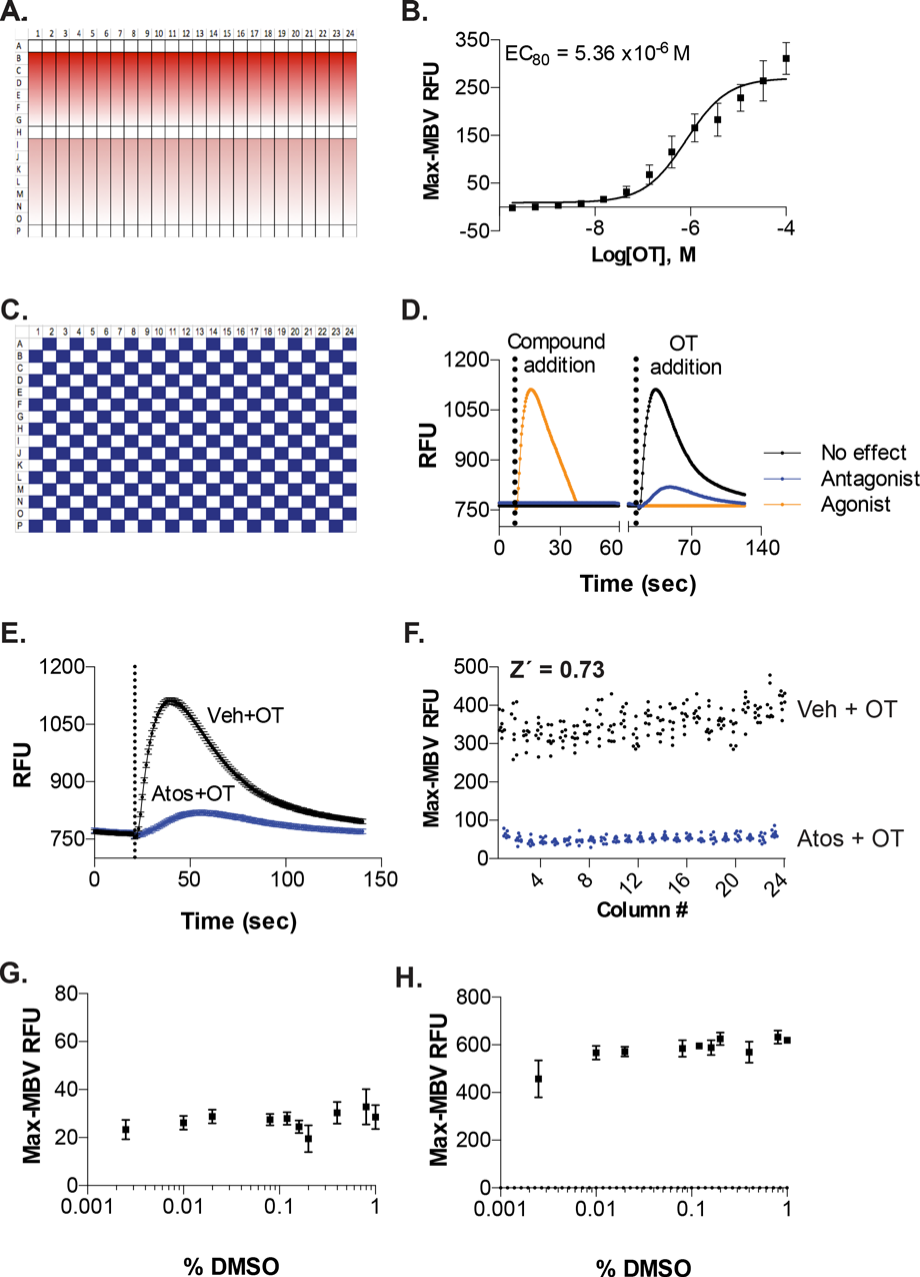
Assay automation and determination of suitability for HTS. A. Platemap used for CRCs performed for OT-induced Ca^2+^-mobilization from UT-myo cells. B. Mean OT CRC from all Ca^2+^-mobilization assays performed for checkerboard analysis and pilot screen (n = 7 independent experiments (batches of primary mouse UT-myo cells) performed in quadruplicate). OT CRCs were used to calculate the OT-EC_80_ for each day of the checkerboard analyses. Mean ± SEM OT EC_80_ is shown. C. Platemap used to perform checkerboard analyses. Every other well received either 0.1% DMSO (final concentration vehicle, white boxes) or 10μM atosiban (blue boxes) for 30 min prior to the addition of OT (EC_80_) to the entire plate. D. Dual-addition assay format for the identification of agonists and antagonists of Ca^2+^-mobilization from UT-myo cells from a single HTS screen. Compound addition (either atosiban or vehicle) followed by 30min incubation at room temperature, then OT addition. E. Representative graph showing the response of UT-myo cells to either vehicle (Veh) or atosiban (Atos) prior to OT-induced Ca^2+^-mobilization. F. The Z´-factor was calculated from checkerboard analyses performed on three separate days. Tolerance of UT-myo cells to the compound solvent, DMSO, added during the “compound addition” (G) prior to OT-induced Ca^2+^-mobilization (H).

We next performed OT concentration-response experiments to establish the concentration of OT needed to reach 80% of its maximal (EC_80_) induced-intracellular Ca^2+^-release from UT-myo cells. A submaximal EC_80_ dose was chosen for our dual-addition assay format because it provides a robust signal window yet allows ‘‘headroom” for identifying potentiators of OT-induced intracellular-Ca^2+^ mobilization. We used the platemap shown in Fig 3A, in which three rows of each plate received only 0.1% DMSO, while the remaining rows received 3-fold dilutions of OT (see Methods for details). The concentration response to OT was used to calculate the OT-EC_80_ for each day of all subsequent assays: 1) checkerboard analyses; 2) pilot screen; and 3) confirmation of hits.

Next, we performed a checkerboard analysis to determine whether our assay could identify agonists of Ca^2+^-mobilization and antagonists of OT-induced Ca^2+^-mobilization from UT-myo cells. Using the platemap shown in Fig 2C, UT-myo cells received either vehicle (Fig 3C, white boxes) or 10uM atosiban (a known OT-receptor antagonist; Fig 2C blue boxes) during the compound addition (Fig 3D). After a 30-minute incubation of the compound, all cells received the EC_80_ dose during the OT addition. Inhibition of OT-induced Ca^2+^-release from UT-myo cells by atosiban was easily distinguished from vehicle (Fig 3E, blue versus black line).

Finally, we calculated the Z´-factor to allow quantification of the suitability of the Ca^2+^-assay for use in a full-scale HTS. The mean±SEM Z´-factor for the assay plates on three separate days was 0.73±0.04, indicating assay robustness and reproducibility (Fig 3F).

In order for an assay to be useful for high-throughput screening, it is necessary to determine the sensitivity of the assay to DMSO, the solvent used for small-molecule compound libraries. Ca^2+^-mobilization from UT-myo cells was unaffected at DMSO concentrations up to 0.4% v/v (Fig 3G), and showed no effect on OT-induced Ca^2+^-mobilization at all concentrations examined (Fig 3H). Thus, DMSO had no effect on Ca^2+^-mobilization or OT-induced Ca^2+^-mobilization at the screening concentrations of 0.1% v/v.

### Pilot Screening for Assay Validation

A pilot screen using three libraries of compounds was performed to assess the suitability of the screen for HTS and potentially identify lead compounds for immediate development. Specifically, the Spectrum Collection consists of 2,000 compounds with a wide range of biological activity and structural diversity: 50% drug components, 30% natural products, 20% other bioactive components; while the NIH Clinical Collection I and II of contains 730 compounds that have a history of use in human clinical trials.

**Fig 4:**
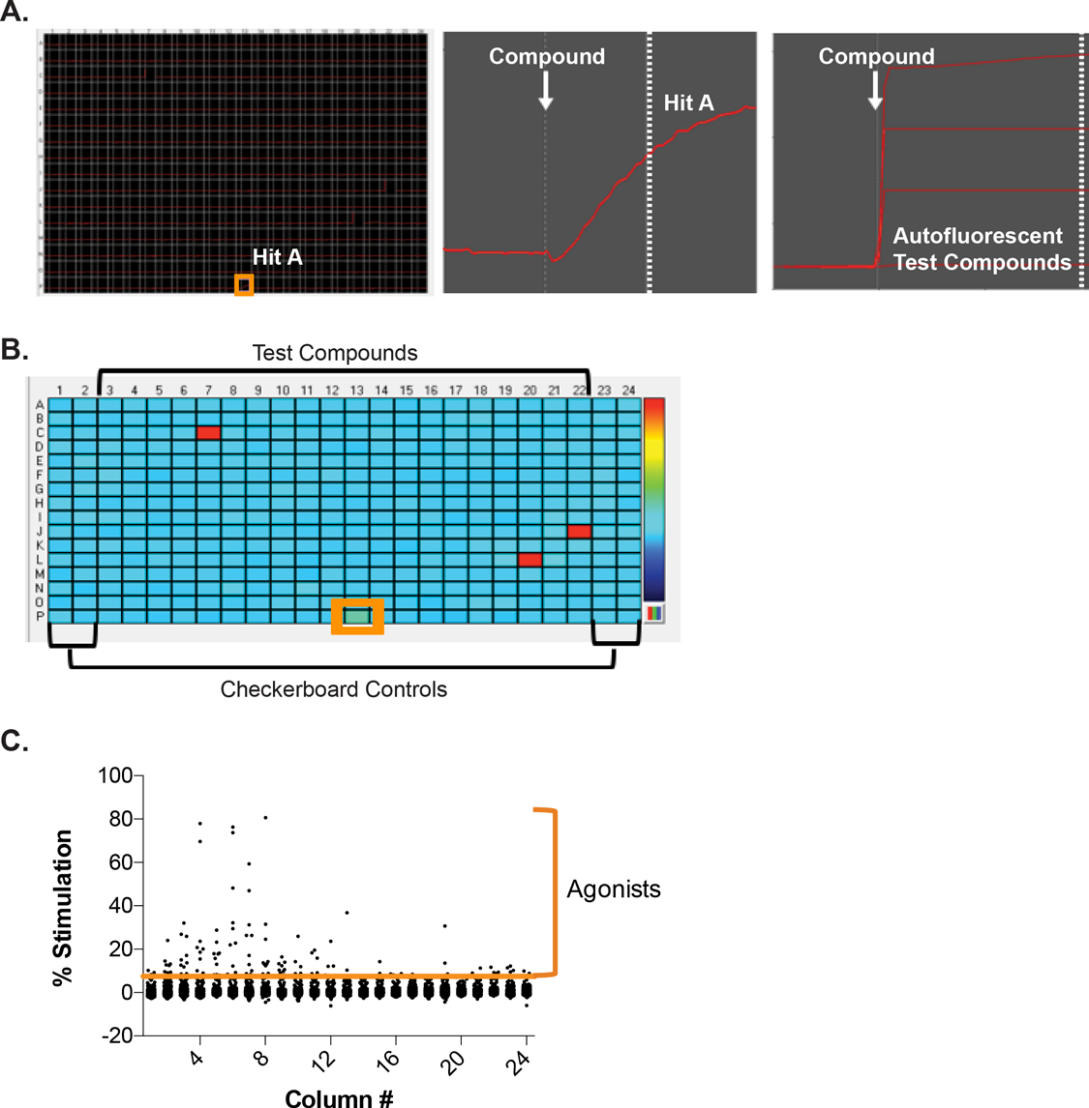
Hit-agonist identification during pilot screen. A, *left panel*. Representative image of realtime monitoring of Ca^2+^-mobilization from UT-myo cells following the compound addition (white arrow, *middle panel*). The two end columns of each plate were used for checkerboard analyses to determine the Z´-factor for each HTS assay, and received either 0.1% DMSO vehicle or 10uM atosiban during the compound addition. The inner 320 wells received 10μM of test compounds. An example of a “Hit”-agonist is highlighted (orange box). A, *right panel*. Examples of relative fluorescent recordings from autofluorescent test compounds. B. Plate heatmap of Ca^2+^-mobilization at the time following compound addition (dashed line, middle panel A). A representative hit-agonist compound (green well) is highlighted by orange box, while autofluorescent compounds are visualized as bright red wells. C. The average cutoff threshold for hit-agonists was 5.85±1.59% stimulation, based on 3*MAD from median (refer to “Materials and Methods” section).

**Fig 5:**
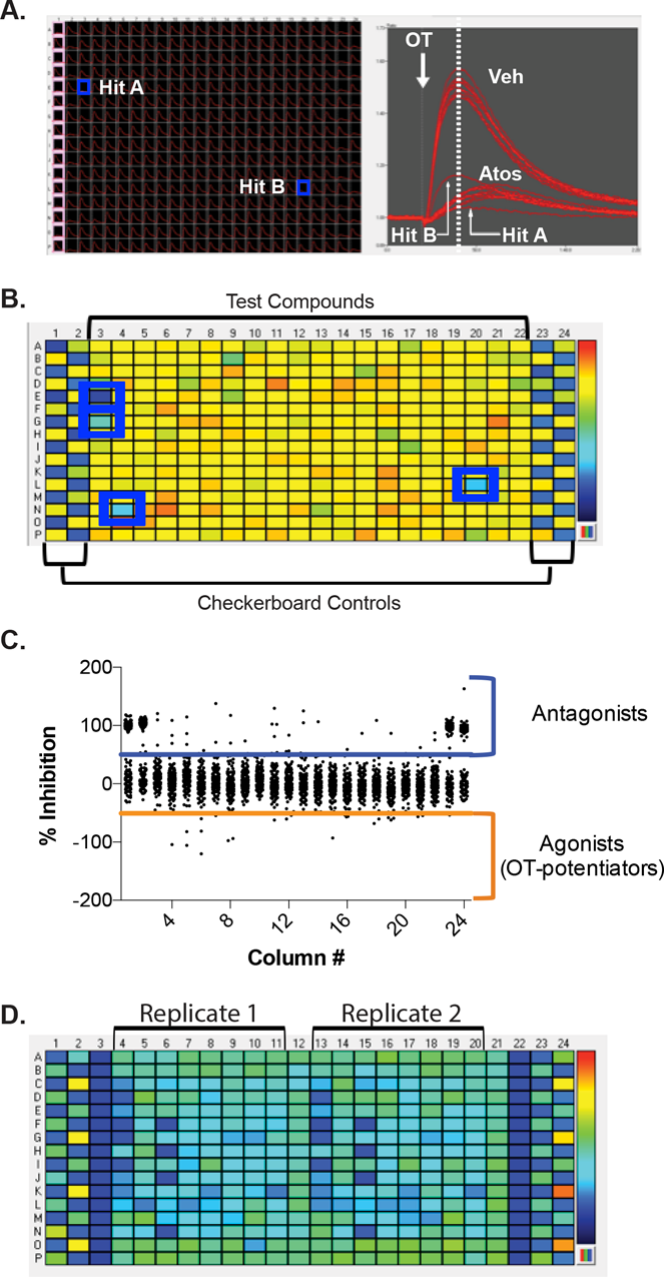
Hit-antagonist identification during pilot screen and confirmation after retesting. A, *left panel*. Realtime monitoring of OT-induced Ca^2+^-mobilization from UT-myo cells. The two end columns (one highlighted in pink) of each plate were used for checkerboard analyses to determine the Z´-factor for each HTS assay. Examples of “Hit”-antagonists are highlighted (blue boxes). A, *right panel*. OT-induced Ca^2+^-mobilization of highlighted wells. White arrow indicates the time of OT addition. B. Plate heatmap of Ca^2+^-mobilization at the time indicated by the dashed line (right panel A). Representative hit-antagonist compounds are highlighted by blue boxes. C. The average cutoff threshold based on 3*MAD from median (refer to “Materials and Methods” section) for “hit”-antagonists was 41.07±3.79% inhibition and “hit”-agonists (OT-potentiators) was 50.02±4.77% activation. D. Representative heatmap of additional Ca^2^-mobilization assays performed to retest hit-agonists and antagonists in duplicate. Veh = vehicle, Atos = atosiban

On each day of screening, the OT EC_80_ value was calculated to determine the ability of test compounds to inhibit this submaximal concentration of OT. On each screening plate, the outer four columns were used for checkerboard analyses to determine the Z´-factor (Figs 4B and 5B). The test compounds filled the remaining 320 wells, and were screened at a nominal final concentration of 10μM.

During the compound addition, most test compounds had no effect on the stimulation of intracellular Ca^2+^-release (Fig 4). However, a small number of compounds stimulated Ca^2+^-mobilization directly (Table 1). Autofluorescent compounds were identifiable by their characteristic immediate (within 1 sec) and sustained effect on fluorescence emission (Fig 4A, right panel compared to middle panel). By highlighting the point at which compounds were added during the Ca^2+^-assay, we were able to quickly identify potential “hit”-agonist compounds (Fig 4B, highlighted by orange box). The average cutoff for active “hit” agonists was 5.85± 1.59% stimulation, which was based on the mean effect of the minimum control and 3 times the SD (Fig 4C).

**Table 1:** Calcium-mobilization HTS parameters.

		Agonists			Antagonists		
Compound Library	# Compounds	# Hit^1^	Hit-Rate	% Confirmed	# Hit^1^	Hit-Rate	% Confirmed
Spectrum	2000	50	2.50	66.7	38	1.90	86.8
NIH Collection I	446	13	2.91	75.0	5	1.12	80.0
NIH Collection II	281	11	3.91	62.5	2	0.71	50.0
Total	2727	74	2.71	66.7 (n = 49)	45	1.65	84.4 (n = 38)
			1.80 confirmed hit-rate			1.39 confirmed hit-rate	

^1^ Autofluorescent compounds excluded.

After the OT addition, there was a noticeable inhibition of OT-induced Ca^2+^-release in UT-myo cells by some of the test compounds examined. A small number of test compounds appeared more effective at inhibiting OT-induced Ca^2+^-release than atosiban (Fig 5A, Hit A compared to atosiban), while other compounds were less or similarly effective as atosiban (Fig 5A, Hit B compared to atosiban). Similar to the identification of agonists, we were able to quickly identify potential “hit”-antagonist compounds (Fig 5B, highlighted by blue boxes) by highlighting the point at which oxytocin was added during the Ca^2+^-assay, The % inhibition was calculated in a similar manner as the % stimulation for each compound. The average cut-off for active “hit”-antagonists was 50.02±4.77% inhibition in OT-induced intracellular Ca^2+^-release at a concentration of 10μM. Table 1 summarizes the number of hits and hit-rates in the pilot screen.

An unexpected observation occurred following the analyses of data collected during the OT addition. As shown in Fig 5C, a second set of test compounds were discovered as potentiators of OT-induced intracellular Ca^2+^-release. In total, 20 test compounds (0.73% of all compounds screened) were found to potentiate OT-induced calcium mobilization following an average cut-off of -50.83±5.55% inhibition after a median -0.84±0.51% inhibition of control OT wells. None of these “hit”-potentiators of OT-induced calcium mobilization overlapped with the “hit”-agonists discovered during the compound addition. Importantly, one small molecule, N-(3-Trifluoromethylphenyl) Piperazine Hydrochloride (TFMPP) was found as a “hit”-potentiator in two different compound screen plates from two different compound libraries (Spectrum and NIH Clinical I Collection) using two different batches of primary mouse UT-myo cells, with an average 48.84±1.75% activation.

Next, we retested hit-compounds (Fig 5D), and found 67% of compounds were confirmed as hit-agonist compounds capable of inducing calcium mobilization in primary mouse UT-myo cells. Furthermore, we confirmed 84% of compounds as hit-antagonists that were effective for inhibiting OT-induced intracellular Ca^2+^-release in UT-myo cells (Table 1).

### Examining the potency of hit-compounds

**Fig 6:**
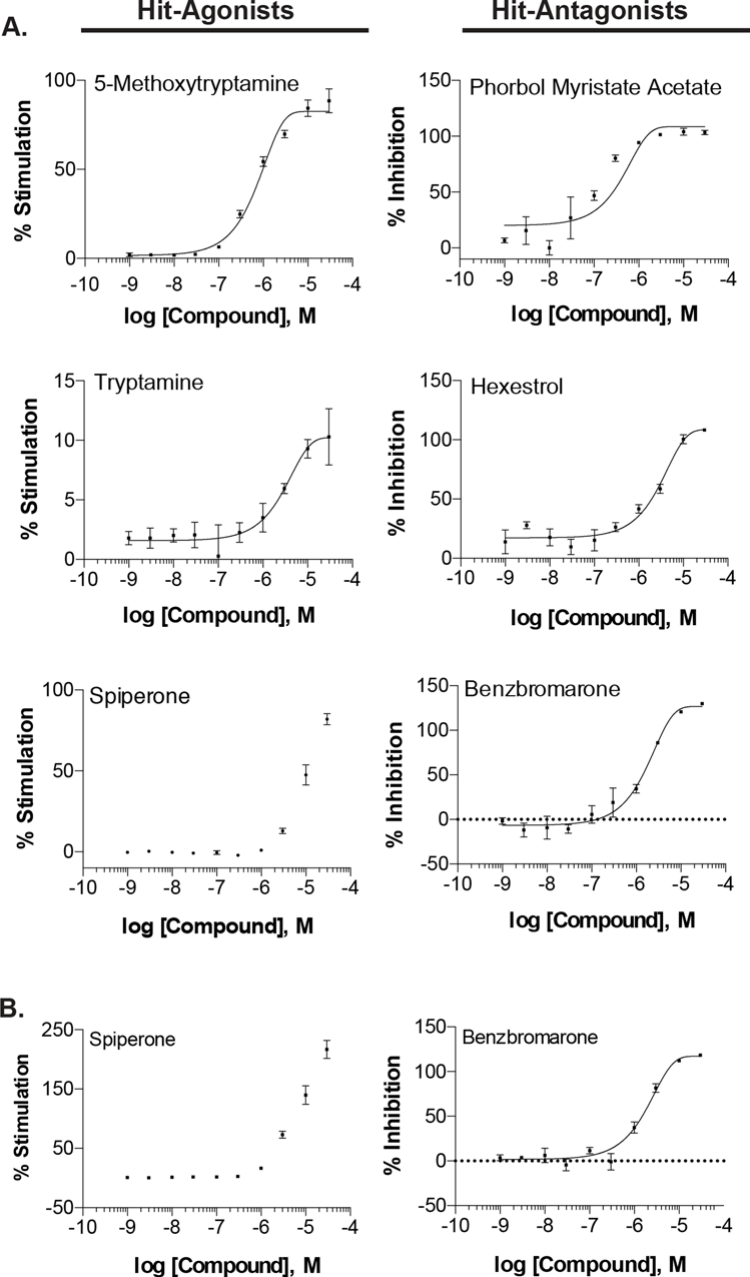
Concentration-response effect of confirmed hit-compounds. A. Representative compound titrations performed to examine the potency of confirmed hit-agonists and hit-antagonists on UT-myo native and OT-induced Ca^2+^-mobilization. B. Additionally, compound titrations for hit-compounds were performed using commercially purchased compounds. A 10-point three-fold titration of confirmed hit-compounds were added during the compound addition of the Ca^2+^-mobilization assay. Data is shown as either mean±SEM %stimulation of Ca^2+^-mobilization or %inhibition of OT-induced Ca^2+^-mobilization. Non-linear regression was used to fit the data.

We examined the potency of the confirmed hit-agonist and antagonist compounds by assaying compounds at three-fold 10-point titrations starting at 30μM. The potency of hit-antagonist compounds was generally greater than that of hit-agonist compounds (Table 2). There were 21 hit-antagonist compounds that demonstrated an EC_50_ less than 10μM, compared to only 7 hit-agonist compounds. Representative concentration-response curves of hit-compounds are shown in Fig 6A. Additionally, we compared the potency of confirmed hit-compounds purchased commercially, to those cherry-picked from the compound library plates. Two representative hit-compounds are shown (Fig 6B vs 6A): spiperone, a hit-agonist and benzbromarone, a hit-antagonist. While the Emax was greater using the spiperone purchased commercially (Emax ± SEM = 217.0 ± 15.24 vs 81.89 ± 3.48) there were no differences in the EC_50_ value (9.30e-06 vs 1.02e-05) between the two sources of spiperone, respectively. Moreover, no differences were noted in either the Emax (118.4 ± 0.94 vs 130.0 ± 0.19) or EC_50_ (1.84e-06 vs 1.90e-06) values obtained using the two sources (commercial vs cherry-picked) of benzbromarone, respectively.

**Table 2:** Concentration-response effect of confirmed hit-compounds.

Compound ID	Compound Name	EC_50_	E_max_ ± SEM
**Agonists**	** **	** **	** **
1388	5-Methoxytryptamine	7.43e-07	90.92 ± 8.1
3005837	Pyrithione Zinc	1.65e-06	17.43 ± 0.88
1150	Tryptamine	3.08e-06	10.29 ± 2.36
41684	Nitazoxanide	4.20e-06	26.9 ± 1.05
54680693	Pyrvinium Pamoate	5.68e-06	22.58 ± 0.99
5649	Valinomycin	6.49e-06	8.72 ± 1.55
5265	Spiperone	1.02e-05	81.89 ± 3.48
34312	Oxycarbazepine	1.10e-05	7.44 ± 0.3
8397	Xanthopterin	1.13e-05	24.78 ± 3.04
3598	Hexachlorophene	1.39e-05	17.79 ± 2.81
3397	Flutamide	2.19e-05	15.23 ± 0.74
5701996	Isoreserpine	3.03e-05	11.53 ± 1.24
3386	Fluoxetine	4.45e-05	21.65 ± 8.32
4993	Pyrimethamine	5.56e-05	85.64 ± 2.4
**Antagonists**			
3037	Dichlorophene	9.44e-07	115.28 ± 7.43
37123	Diflubenzuron	9.93e-07	35.15 ± 2.99
52897276	7-Desacetoxy-6,7-Dehydrogedunin	1.07e-06	33.83 ± 6.85
16682730	Phenylmercuric Acetate	1.13e-06	112.88 ± 3.41
6083	Adenosine 5’-monophosphate	1.16e-06	49.74 ± 9.49
27924	Phorbol Myristate Acetate	1.27e-07	103.43 ± 1.94
10205	Plumbagin	1.50e-06	114.4 ± 3.67
3352	Fipronil	1.70e-06	80.65 ± 8.95
3606	Hexestrol	3.36e-06	108.18 ± 0.72
2333	Benzbromarone	1.91e-06	130.03 ± 0.19
6377243	Obtusaquinone	2.83e-06	114.15 ± 8.08
72385	Nonoxynol-9	4.48e-06	123.35 ± 5.55
3492326	Dihydromunduletone	5.03e-06	122.17 ± 4.87
452550	Tyrothricin	7.19e-06	139.95 ± 13.1
9556529	Oxiconazole Nitrate	7.44e-06	80.33 ± 6.91
3651377	MK-886	7.92e-06	152.43 ± 0.93
2378	Bifonazole	8.66e-06	61.95 ± 2.77
4499	Nisoldipine	9.43e-06	144.42 ± 1.97
2330	Benzalkonium Chloride	9.82e-06	115.68 ± 4.9
3503	Gossypol	1.00e-05	119.51 ± 0.7
73357	Gramicidin	1.08e-05	63.77 ± 4.03

### Lead hit generation using functional annotation analyses

We performed functional annotation analyses in order to rank confirmed hit-antagonists into leads in order to limit the number of hits examined in our *ex vivo* isometric contractility assay. The MeSH pharmacological classification of each confirmed hit-compound was obtained from PubChem. As shown in Table 3, the majority of compounds have unknown MeSH pharmacological classification, despite our pilot screen containing well-annotated compound libraries. Surprisingly, a number of our hit-agonist compounds are anti-psychotic agents (3), dopamine antagonists (3), and adrenergic uptake inhibitors (2). As expected, many of our hit-antagonist compounds are known vasodilator agents (3), antihypertensive agents (2), bronchodilator agents (2), Ca^2+^-channel blockers (2) cardiotonic agents (2), all of which are known to affect intracellular Ca^2+^-levels in smooth muscle cells. Our screen also identified a known tocolytic agent, fenoterol. Interestingly, several of our confirmed hit-antagonist compounds have anti-inflammatory or immunological pharmacological classifications: anti-Infective agents, local (3), immunologic adjuvants (2), antifungal (2), and anti-bacterial (2) compounds.

**Table 3:** MeSH pharmacological classifications containing multiple confirmed hit-compounds.

MeSH Pharmacological Classification	# Compounds
**Agonists**	
Unknown	6
Antineoplastic	3
Antipsychotic Agents	3
Dopamine Antagonists	3
Adrenergic Uptake Inhibitors	2
Anti-Infective Agents	2
Antidepressive Agents, Second-Generation	2
Antineoplastic	2
Insecticides	2
Ionophores	2
Serotonin Uptake Inhibitors	2
Uncoupling Agents	2
**Antagonists**	
Anti-Infective Agents, local	3
Vasodilator	3
Adjuvants, Immunologic	2
Antifungal Agents	2
Antihypertensive Agents	2
Bronchodilator Agents	2
Calcium-Channel Blockers	2
Cardiotonic Agents	2
Contraceptive Agents	2

The compound identification (CID) number of each confirmed hit compound was uploaded into BioActivity SAR in PubChem, followed by clustering of Compound Activity and Active BioAssay Defined Protein Targets. Shown in Table 4 are the target proteins of prior HTS bioassay studies in which multiple of our confirmed hit compounds were found to be active. Similar to the MeSH pharmacological classifications, a number of hit-agonist compounds have been shown to target the adrenergic, serotonin and dopamine receptors.

**Table 4:** Target proteins identified by multiple confirmed compounds.

Target Proteins	# Compounds
**Agonists**	
Cytochrome P450, family 1, subfamily A, polypeptide 2	8
Alpha-2C adrenergic receptor	4
5-hydroxytryptamine receptor 2B	3
Cytochrome P450 2D6	3
E3 ubiquitin-protein ligase Mdm2 isoform MDM2	3
Nuclear factor erythroid 2-related factor 2 isoform 1	3
5-hydroxytryptamine receptor 4	2
5-Hydroxytryptamine receptor 5-HT7	2
Bromodomain adjacent to zinc finger domain 2B	2
Chain A, Jmjd2a Tandem Tudor Domains In Complex With A Trimethylated Histone H4-K20 Peptide	2
Chromobox protein homolog 1	2
Histamine H1 receptor	2
Microtubule-associated protein tau	2
Nuclear receptor ROR-gamma	2
Platelet-activating factor acetylhydrolase precursor	2
Potassium voltage-gated channel subfamily KQT member 2	2
Prolyl endopeptidase-like	2
Putative recombination protein RecB	2
Serine/threonine-protein kinase mTOR	2
**Antagonists**	
Vitamin D3 receptor isoform VDRA	3
Glyceraldehyde-3-phosphate dehydrogenase	2
Nuclear receptor ROR-gamma	2
Tumor protein p53	2

### Testing confirmed hit antagonists for tocolytic ability

Based on the percent inhibition and functional annotation analyses, we chose to focus the remainder of the studies on antagonist compounds. We selected 4 confirmed hit-compounds (benzbromarone, dipyridamole, fenoterol HBr and nisoldipine; Table 5) to determine whether any of these inhibitors of UT-myo cell Ca^2+^-release could prevent uterine myometrial contractions. A well-established *ex vivo* isometric contractility assay was used as a secondary screen to examine the effect of hit-antagonist compounds on frequency, amplitude and AUC [25–31].

**Table 5:** Compounds selected for *ex vivo* uterine myometrial organ bath contractility studies.

Compound	AVG % Inhibition Ca^2+^-Assay^1^	MeSH Pharmacological Category	Target protein
Atosiban	99.67	Hormone antagonist; Tocolytic agent	OTR, V1aR, V1bR, V2R
Benzbromarone	100.22	Uricosuric Agent	P450, PPARy, PNR, MCL1, GP120,KCNJ1,SKA, TNF, S1P1
Dipyridamole	128.11	Phosphodiesterase Inhibitors; Platelet Aggregation Inhibitor; Vasodilator Agent	PDE3 and 10, P450, ENT1, M1R
Fenoterol Hydrobromide	47.66	Adrenergic beta-2 receptor agonist; Bronchodilator Agent; Sympathomimetic; Tocolytic Agent	Beta-2AR, DRD1, EP2
Nisoldipine	63.49	Antihypertensive Agent; Calcium Channel Blocker; Vasodilator Agent	KCNH2

^1^ Values obtained from primary screen and retesting at 10μM concentration

**Fig 7:**
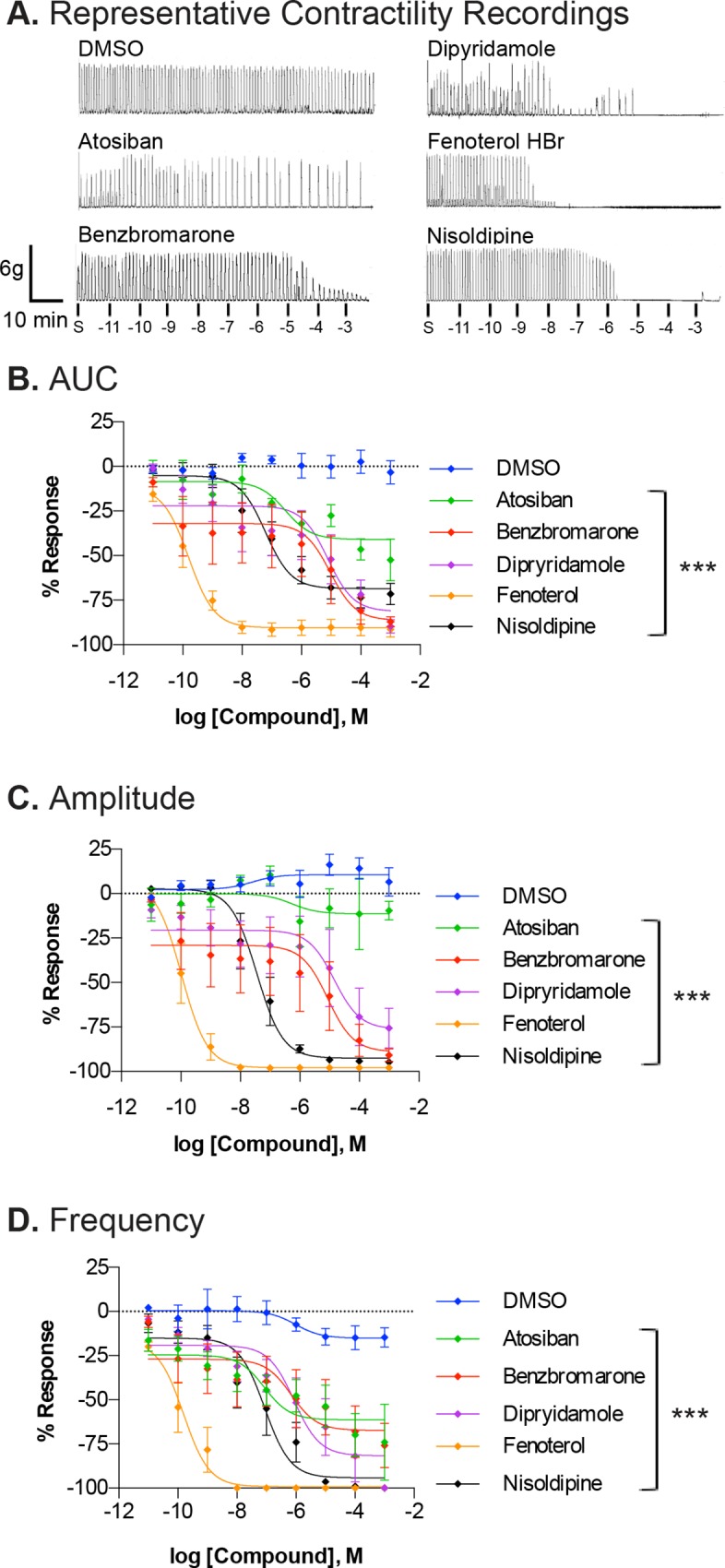
Effect of confirmed hit-compounds on *ex vivo* uterine myometrial contractility. A. Representative recordings of spontaneous contractile activity prior to treatment with increasing concentrations (10pm to 1mM) of either DMSO (vehicle control), atosiban, benzbromarone, dipyridamole, fenoterol HBr or nisoldipine. Isometric tension recordings were analyzed for AUC (B), amplitude (C) and frequency (D) of contractions. Data is shown as mean±SEM % response from baseline (spontaneous contraction) from 5–8 different uterine strips from different mice. Non-linear regression was used to fit the data. Significant (***p<0.0001) difference between each fit line and DMSO is indicated. S = spontaneous contractility

Spontaneous myometrial contractions were recorded (measured in grams, g, of tension) for 10min, followed by the addition of cumulative doses of vehicle, atosiban, benzobromarone, dipryidamole, fenoterol HBr or nisoldipine (10pM to 1mM; 10min per dose) as shown in representative recordings **(** Fig 7A). All compounds examined significantly (p<0.001) inhibited contractile activity (Fig 7B–7D) at different IC_50_ values (Table 6). Fenoterol and nisoldipine were found to be the most potent (IC_50_ range = 0.1–0.157 nM and 35.2–87.2nM, respectively) compounds examined based on all parameters: AUC, amplitude and frequency. Fenoterol and nisoldipine also had the greatest efficacy (E_max_: -90.47 to -99.12%, and -68.52 to -94.13%, respectively).

**Table 6:** Capacity of compounds to inhibit *ex vivo* uterine myometrial contractility.

		AUC		Amplitude		Frequency	
Compound	N	IC_50_	E_max_	IC_50_	E_max_	IC_50_	E_max_
Atosiban	5	2.875e-07	-40.93%	5.340e-07	-11.36%	8.499e-08	-61.31%
Benzbromarone	5	9.185e-06	-86.35%	8.677e-06	-88.95%	6.932e-07	-67.29%
Dipyridamole	8	7.694e-06	-81.37%	1.871e-05	-81.49%	8.64e-07	-81.67%
Fenoterol HBr	7	1.577e-10	-90.47%	1.056e-10	-97.86%	1.549e-10	-99.12%
Nisoldipine	8	6.134e-08	-68.52%	3.528e-08	-92.14%	8.725e-08	-94.13%

In order to relate the results of our Ca^2+^-mobilization assay to results from our secondary *ex vivo* contractility assay, we compared the inhibitory effects of the compounds between these two assays. We found that the rank order potencies of compounds obtained from the Ca^2+^-mobilization microtiter plate assay did not necessarily correlate with our secondary contractility assay (Table 7). While fenoterol was the least inhibitory compound in the Ca^2+^-mobilization assay, it was the most potent compound examined in the isometric contractility assay. Conversely, benzbromarone was one of the least potent compounds to inhibit myometrial contractility, but was selected for examination in this secondary assay based on its high inhibitory capacity in the Ca^2+^-mobilization assay.

**Table 7:** Comparison of inhibitory capacity of compounds between calcium-mobilization and *ex vivo* uterine myometrial contractility assays.

		High-throughput Ca^2+^ Assay			*Ex Vivo* Isometric Contractility (AUC)	
Compound	N	IC_50_	E_max_	N	IC_50_	E_max_
Atosiban	3	2.66e-07	-113.0%	5	2.9e-07	-40.93%
Benzbromarone	3	1.91e-06	-130.0%	5	9.2e-06	-86.35%
Dipyridamole	3	1.50e-05	-135.2%	8	7.7e-06	-81.37%
Fenoterol HBr	3	2.25e-05	-37.76%	7	1.6e-10	-90.47%
Nisoldipine	3	9.43e-06	-144.4%	8	6.1e-08	-68.52%

## Discussion

The identification of novel tocolytic or uterotonic compounds remains of paramount importance considering off-target side effects and inefficacy of existing treatments. We report for the first time a robust and reliable uterine myometrial calcium mobilization assay for HTS. The assay was validated for HTS by meeting a series of rigorous performance benchmarks: 1) DMSO-tolerance; 2) assay robustness and reproducibility based on a Z´ = 0.73; 3) a pilot screen that demonstrated the suitability of the assay for HTS, the feasibility of using primary mouse uterine myometrial cells for future large-scale HTS, and identified hit-compounds for immediate testing in a secondary screen; and 4) assay capability of reporting dose-dependent stimulation of calcium-mobilization and inhibition of OT-induced calcium-mobilization. Using a secondary *ex vivo* assay, we established that four representative hit-antagonists are potent inhibitors of uterine contractility. Collectively, these findings demonstrate the power of the Ca^2+^-mobilization assay to identify tocolytic compounds.

The assay described here is the first Ca^2+^-mobilization assay in a 384-well format to screen primary uterine myometrial cells. Our HTS assay identified both agonists of Ca^2+^-mobilization and antagonists of OT-induced Ca^2+^-mobilization in uterine myometrial cells in a single-screen. Unexpectedly, it also revealed potentiators of OT, *i*.*e*. agonists of OT-induced Ca^2+^-mobilization. Our assay was found to be robust (Z´-factor = 0.73) and suitable for use in a full-scale HTS campaign to correctly distinguish “hits” from non-hits. Furthermore, our pilot screen using well-annotated compound libraries allowed us to assess assay quality and predict its usefulness in a HTS campaign. Based on the calculated % activation and % inhibition thresholds, the calculated hit-rate prior to confirmation testing was 2.71% for agonists and 1.65% for antagonists, respectively. Large-scale screening for the discovery of new uterotonics or tocolytics is sorely needed. Thus, the robust HTS-ready assay described here may provide a reliable method for identification of novel modulators of uterine Ca^2+^-mobilization and myometrial contractility.

The cut-off thresholds used to identify hit-compounds during the pilot screen were determined empirically using industry-standard, statistics-based selection criteria. The average hit-cutoff for hit-agonists was 5.85% stimulation. On average, identified hit-agonists were weaker (lower E_max_ and EC_50_ values) compared to identified hit-antagonists. Despite this, 49 (or 67%) of the hits were confirmed during retesting, and 7 of those had EC_50_ values ≤10 uM. In a large-scale HTS, where presumably more potent agonists (and antagonists) will be identified, compounds exhibiting more potent activity can be prioritized over weaker modulators.

False-positives can be a major problem during HTS. Autofluorescence of some test compounds resulted in 22 false positives in the pilot screen. Other false positives were removed during retesting of hit-antagonists as well as titration studies to examine potency. During large-scale HTS campaigns, it is necessary to utilize counterscreen assays in order to remove false-positives and prioritize hit-compounds. Common counterscreen assays include: target or cell selectivity, or compound cytotoxicity. Application of these assays will be important to determine whether any of the hit-compounds identified in this study are: 1) artifacts of detection, 2) specific agonists of uterine Ca^2+^-mobilization or antagonists of OT-induced uterine Ca^2+^-mobilization or 3) cytotoxic (relevant to antagonists only). To this end, we have begun developing a comparative screen for UT-myo selectivity using the primary Ca^2+^-mobilization assay described in this study and primary mouse aorta smooth muscle cells (AOSMCs) [41]. Six hit-agonists were capable of inducing intracellular Ca^2+^-release from AOSMCs, while 7 hit-agonists have been identified as UT-myo selective and have an EC50≤10μM: 5-methoxytryptamine, tryptamine, nitazoxanide, oxycarbazepine, xanthopterin, fluoxetine and pyrimethamine (from Table 2). In order to test hit-antagonists for uterine-selectivity, we chose to use U46619, instead of OT, to induce intracellular Ca^2+^-release from AOSMCs since it is established as the most potent agonist of vascular SMCs. Using this strategy, we have identified 6 promiscuous hit-antagonists that are *not* UT-myo selective: dichlorophene, 7-Desacetoxy-6,7-Dehydrogedunin, plumbagin, tyrothricin (from Table 2). In future studies, we hope to develop comparative counterscreen assays using the primary Ca^2+^-mobilization assay, described in this study, using mesenteric artery SMCs and fetal ductus arteriosus SMCs, given that these SMC-types (along with AOSMCs) are the most relevant off-targets of current uterotonic and tocolytic therapeutics.

Functional annotation analyses of confirmed agonists and antagonists provided insight into molecular target pathways and pharmacological classes of agents affecting uterine myometrial Ca^2+^-mobilization. Our pilot screen identified a number of pharmacologic classifications and targets of current uterotonic (alpha-adrenergic receptor agonist) and tocolytic (vasodilators, calcium channel blockers, and beta-2 adrenergic receptor agonists) agents. However, we anticipate that other members of pharmacologic classes of compounds that were uncovered in our analysis could be explored for their potential uterotonic and tocolytic capabilities. To this end, most currently used tocolytics were initially developed for pain or cardiovascular problems, but were later found to be effective tocolytics [13]. Moreover, a recent study identified the inward rectifier potassium channel Kir7.1 as a critical regulator of myometrial cell membrane potential and contractility. It will be important to determine if any of the agonists of calcium signaling identified in the present study act on Kir7.1 to regulate calcium entry [42].

Testing hit-compounds in a tissue assay serves as an excellent transitional screening system between cell-based assays and *in vivo* models. To this end, *ex vivo* uterine myometrial contractility assays are a well-established and ideal model for testing the therapeutic capacity to regulate tissue contractility. The results obtained from the uterine myometrial contractility assay showed that all 4 hit-compounds identified from our pilot screen were able to inhibit *ex vivo* uterine contractions. The IC_50_ values obtained from the contractility assay were not highly correlated with those obtained from the Ca^2+^-mobilization assay. We attribute this discrepancy to differences between the tissue strips used in our secondary screen compared to isolated single-cells used in our primary screen. Despite these differences, both screening approaches identified significant inhibitory effects on uterine contractility; their combined use provided additional strength for small molecule screening. Some of these compounds have previously been explored for their inhibitory potential on uterine contractility. Dipyridamole was examined with other phosphodiesterase inhibitors to determine whether they could potentiate 5-hydroxytryptamine inhibition of pig myometrial contractility [43]. Nisoldipine has been previously reported to inhibit spontaneous contractility of non-pregnant human myometrium, as well as OT-induced rat myometrial contractions [44]. The IC_50_ of nisoldipine reported in that study (6±0.09 μM) was less potent in non-pregnant human myometrial samples compared to what we report (range: 0.04–0.09 μM) for pregnant mouse myometrial samples. Finally, fenoterol has been investigated and shown clinically to inhibit uterine activity, by up to 30%, in women with OT-induced labor [45]. While fenoterol has been shown to be an effective agent for treatment of preterm labor, its use as a tocolytic in women was terminated due to maternal adverse effects [46].

Collectively, this study developed and validated a robust dual-addition assay for HTS to identify agonists and antagonists of Ca^2+^-mobilization in UT-myo cells. HTS was shown to be an effective tool for discovering small molecular compounds regulating uterine myometrial Ca^2+^-mobilization. Furthermore, inhibition of myometrial contractions by 4 compounds identified by our HTS suggests that small molecules have the potential to regulate uterine contractility. Together, these data highlight a promising role for large-scale HTS for identifying small molecule molecular modulators and molecular targets of myometrial Ca^2+^-mobilization, which have high therapeutic potential for women with preterm labor or postpartum hemorrhage/uterine atony.
